# First pre-filled pen device with highly purified human menopausal gonadotropin (HP-hMG, Menopur) in liquid is shown to be bioequivalent to powder for reconstitution 

**DOI:** 10.5414/CP204040

**Published:** 2021-10-08

**Authors:** Daniël M. Jonker, Manuela Koch, Per Larsson, Arjun Ravi, Birgitte Buur Rasmussen, Runa Speer, Bernadette Mannaerts

**Affiliations:** 1 International PharmaScience Center, Ferring Pharmaceuticals, Copenhagen, Denmark,; 2 Nuvisan GmbH, Neu-Ulm, and; 3 CRS Clinical Research Services GmbH, Berlin, Germany

**Keywords:** bioequivalence, highly purified hMG, serum FSH, serum hCG

## Abstract

Objective: To determine whether serum human follicle-stimulating hormone (FSH) levels after single subcutaneous dosing of highly purified human menopausal gonadotropins (HP-hMG) in a liquid formulation and a powder formulation are bioequivalent. Materials and methods: This was a randomized, two-way, crossover, single-dose, bioequivalence trial comparing Menopur liquid injected by pre-filled pen, with Menopur powder injected by conventional syringe and needle. The primary endpoints were AUC_t_ and C_max_ of baseline-adjusted FSH. Pituitary-suppressed, healthy women were administered single subcutaneous injections of 450 IU Menopur liquid (600 IU/0.96 mL) and 450 IU Menopur powder (by 2 subcutaneous injections of 225 IU in 1 mL) in a randomized order. The pharmacokinetic parameters of FSH and human chorionic gonadotropin (hCG) were assessed by non-compartmental methods with adjustment for endogenous pre-dose levels. Results: In total, 76 women were randomized, and 56 completed the trial. The mean FSH and hCG serum concentration-time profiles were comparable between the two HP-hMG formulations. The geometric mean ratios and 90% confidence intervals of FSH for HP-hMG liquid versus HP-hMG powder were 1.12 (1.0562 – 1.1889) for AUC_t_ and 1.17 (1.0946 – 1.2490) for C_max_, showing that the two formulations were bioequivalent. The incidence and severity of adverse events were similar between the two preparations, and both preparations were well tolerated. Conclusion: The 90% CIs for the geometric mean ratios of serum FSH AUC_t_ and C_max_ were both within 0.8000 – 1.2500, thus the two formulations are bioequivalent.

**What is known about this subject**

- For several decades, HP-hMG (Menopur) has been used for the treatment of infertility; its efficacy and safety compared to other gonadotropins have been consistently demonstrated in several prospective, randomized controlled trials and meta-analyses [1, 2].
- Menopur powder for reconstitution is available in multi-dose and single-dose formulations. Up to 3 single-dose vials (each containing 75 IU) may be dissolved into 1 mL solvent for administration.
- Recently, and for the first time, Menopur has been successfully formulated in a stable, ready-to-use solution for injection, for administration by a pre-filled pen.

**What this study adds**

- The new HP-hMG solution for injection in a pre-filled pen will deliver the efficacy and safety of Menopur in a convenient delivery device.

## Introduction 

Exogenous gonadotropins have been used to treat infertility for ~ 60 years, first for ovulation induction in anovulatory women and later also in association with assisted reproductive technologies (ART) [3]. At present, highly purified human menopausal gonadotropins (HP-hMG) and recombinant human follicle stimulating hormones (rFSH) are the two most frequent gonadotropin products utilized for infertility treatment either as monotherapies or as mixed protocols. HP-hMG contains an equal ratio of FSH bioactivity and luteinizing hormone (LH) bioactivity, the latter mainly due to human chorionic gonadotropin (hCG) derived from the urine of postmenopausal women, namely 10 IU hCG per 75 IU of urinary FSH [4]. HP-hMG contains also low concentrations of LH, having only a small contribution to the total LH bioactivity. 

The pharmacokinetics of FSH in menotropins has been well-described. In pituitary-suppressed healthy women, serum FSH concentrations reach a maximum after 22 – 27 hours, and the terminal half-life is 39 – 45 hours after a single subcutaneous dose [5]. The bioavailability is similar after subcutaneous and intramuscular administration [6] and was determined to be 74% after intramuscular administration [7]. Regardless of their purity, the pharmacokinetic (PK) profile of menotropins is dose-proportional [5, 8]. There is a paucity of published data on the pharmacokinetics of hCG in HP-hMG after subcutaneous or intramuscular administration, but after intravenous administration, hCG has a terminal half-life of 12 – 15 hours, compared to 21 – 22 hours for FSH in the same study [9]. 

In in vitro fertilization (IVF) patients, the efficacy and safety of HP-hMG in comparison to rFSH has been well-established in large prospective randomized trials both in a long gonadotropin-releasing hormone (GnRH) agonist protocol [10] and in a GnRH antagonist protocol [11, 12]. Meta-analyses have shown that pregnancy and live birth rates are slightly higher following HP-hMG rather than rFSH treatment [1, 13, 14, 15], the difference of which may be related to the FSH and hCG bioactivity of HP-HMG. 

Both hMG and rFSH preparations were initially developed as freeze-dried powder in vials with solvent for reconstitution. To improve the convenience of drug administration, hMG preparations were further developed into multi-dose vials, and rFSH preparations were developed to liquids in different strengths and later in cartridges to be used with a pen-injector [16]. The development of hMG liquid was initially complex due to its impurities, but further purification processes resulted in highly purified hMG allowing for the development of HP-hMG liquid in a pre-filled pen designed for self-administration. Today, there is already abundant experience with pen devices delivering rFSH both for patients undergoing ovarian stimulation prior to IVF or intracytoplasmic sperm injection (ICSI) [17] and for anovulatory women undergoing ovulation induction [18]. In general, patients appreciate pen devices more than conventional syringes as their smaller needles are less painful and they are easier to handle with a reduced number of steps to prepare and administer the drug [19]. Moreover, fewer and easier steps are likely to reduce injection errors and contribute to patient confidence [20]. 

The main question related to this new drug device is whether the PK profile is similar to the PK profile for the existing preparation. Demonstration of bioequivalence between the two treatments would support the idea that IVF patients can interchange pen and powder without expecting any impact on their ovarian response. In the current randomized, single-dose crossover trial, the bioequivalence of HP-hMG (Menopur, Ferring Pharmaceuticals, Copenhagen, Denmark) in two different formulations and presentations, namely HP-hMG liquid injected by a pre-filled pen and HP-hMG powder injected by a conventional syringe were compared in healthy female subjects of reproductive age. 

## Materials and methods 

This was an open-label, randomized, 2-way crossover, single-dose trial conducted at Nuvisan GmbH (Neu-Ulm, Germany) and Clinical Research Services (Berlin, Germany) between November 2018 and September 2019. Regulatory permission to perform the trial was obtained from Bundesinstitut für Arzneimittel und Medizinprodukte (BfArM) on 21 June 2018, in accordance with applicable regulatory requirements. All ethical and regulatory approvals were available prior to a subject being exposed to any trial-related procedure, including screening tests for eligibility. All subjects gave written informed consent before participating in the study. The study was conducted in compliance with the Declaration of Helsinki and according to the European Community Note on Good Clinical Practice for Trials on Medicinal Products in the European Community [21]. 

### Study population 

This study included healthy female subjects aged 20 – 40 years with a body weight ≤ 100 kg and a body mass index (BMI) of 18.5 – 29.9 kg/m^2^. Included subjects were using hormonal contraceptives (oral tablets or vaginal ring) prior to the trial, but discontinued their hormonal contraceptives before enrolment in the trial. To exclude pregnancy, all subjects used an effective non-hormonal method of contraception during the entire trial until the follow-up visit. At screening, all subjects had negative serology for hepatitis B surface-antigen, hepatitis C antibodies, and HIV-1 and HIV-2 antibodies, and negative urine drug screen and alcohol breath test. Main exclusion criteria were presence or history of clinically significant diseases, any clinically significant abnormal laboratory value, history of (or current) endocrine abnormalities such as hyperprolactinemia, polycystic ovary syndrome, and any evidence of ovarian dysfunction, contraindications for the use of oral contraceptives or gonadotropins. 

### Study drugs 

Female subjects received a single dose of HP-hMG (Menopur) from each of two formulations, 450 IU HP-hMG liquid and 450 IU HP-hMG powder, in a randomized order. HP-hMG liquid was provided as a pre-filled pen containing a 3-mL cartridge with solution for injection of 600 IU HP-hMG/0.96 mL containing 600 IU of FSH bioactivity and 600 IU of LH bioactivity. The excipients contained in the HP-hMG liquid are methionine, arginine hydrochloride, phenol, polysorbate 20, sodium hydroxide/hydrochloric acid, and water for injection. The pre-filled injection pen is a non-sterile disposable device with a 31G needle (8 mm long with 6-bevel geometry) delivering doses from 6.25 to 450 IU in increments of 6.25 IU. For administration of HP-hMG liquid, the pen was set at its maximum dose of 450 IU, and 0.72 mL was injected subcutaneously into the lower part of the abdomen. HP-hMG powder was provided as vials with powder and vials with solvent. Prior to administration, 2 sets of 3 vials containing 75 IU each were serially reconstituted with 1 mL/set of 0.9% sodium chloride for injection using a sterile syringe with a reconstitution needle. Subsequently, a syringe with an injection needle (27G) was used to inject 2 times 1 mL subcutaneously into the lower part of the abdomen. 

### Study procedures 

In order to suppress endogenous FSH during the trial, all women received 3 injections of a depot formulation of a GnRH agonist (Decapeptyl, Ferring Pharmaceuticals, Copenhagen, Denmark) that was administered 28 days before the first HP-hMG administration, 7 days before the first HP-hMG administration, and between treatment period 1 and 2, when all samples of period 1 were collected. Whether women were appropriately down-regulated was assessed prior to the first HP-hMG administration and prior to the second HP-hMG administration; it was necessary to demonstrate that serum FSH and estradiol were ≤ 5 IU/L and ≤ 50 pg/mL, respectively. All down-regulated women were randomized to one of the two treatment sequences and received 450 IU of one of the two formulations of HP-hMG on day 1 in treatment period 1 and the alternate formulation on day 1 in treatment period 2. The time interval between the two injections was 19 – 21 days. Safety was evaluated from the time informed consent was signed until the end-of-trial visit, occurring on day 12 – 19 in treatment period 2 in subjects completing the trial. The safety evaluation consisted of adverse events (including serious adverse events and adverse events leading to discontinuation), clinical chemistry, hematology, and urinalysis evaluations as well as vital signs and ECG measurements. In addition, physical examinations, transvaginal ultrasounds, and injection-site reactions were assessed. Injection-site reactions assessed were erythema, pain, pruritus, edema, and bruising, immediately after administration, 0.5, and 24 hours after each administration of HP-hMG. Adverse events were categorized according to the Medical Dictionary for Regulatory Activities coding system version 21.0 and analyzed by severity and relationship to drug. Adverse events with onset after start of first administration of HP-hMG were considered treatment-emergent. 

Blood samples for assessment of immuno-active FSH and immuno-active hCG were collected pre-dose (–1, –0.5 hours and just prior to dosing) and post-dose at 4, 8, 12, 16, 20, 24, 28, 32, 36, 40, 44, 48, 72, 96, 120, 168, and 216 hours. The blood samples were allowed to clot followed by centrifugation, and the serum fraction was collected and stored at –70 °C until analysis. Serum samples were analyzed using highly sensitive and specific electrochemiluminescence immunoassays. The lower limit of quantification of the FSH and hCG assays were 1.47 mIU/mL and 0.5 mIU/mL, respectively. The total precision (CV) was within 5% for both assays during pre-study validation, and the in-study validation revealed a total precision (CV) of < 6.6% for FSH and < 7.9% for hCG at all QC concentration levels. Long-term stability of FSH in serum was shown for up to 753 days with a maximal bias of –3.7% at –20 °C and of –4.8% at –70 °C. For hCG in serum, long-term stability was shown for up to 484 days with maximal bias of –19.5% at –20 °C and –16.7% at –70 °C. Both assays were designed to measure only intact FSH and hCG molecules without cross-reactivity to free subunit. In the FSH assay, HP-hMG liquid was used as an analytical standard, where its biological FSH activity, expressed in IU/L, was used as the nominal content for concentration. For the hCG assay, a urine derived WHO-standard (NIBSC code 07/364) for immunoassay was used as analytical standard. 

### Study endpoints and pharmacokinetic evaluation 

The primary PK endpoints were AUC_t_ and C_max_ of baseline-adjusted serum FSH concentrations. Secondary PK endpoints were AUC_inf_, t_max_, T_1/2_, λ_z_, CL/F, and V_z_/F for FSH, and AUC_t_, AUC_inf_, and C_max_ for hCG, all calculated from baseline-adjusted serum concentrations. Secondary safety endpoints included type, frequency, and intensity of adverse events, change from baseline in vital signs, 12-lead electrocardiogram (ECG), clinical chemistry, hematology, and urinalysis. 

The PK analysis-set comprised 56 women who were dosed in both treatment periods. A total of 6 women were excluded from the hCG analysis because they had falsely elevated hCG concentrations in pre-dose and post-dose samples of both treatment periods, most probably due to heterophile antibody interference (see Discussion). 

Before calculation of PK parameters, the serum concentrations were baseline-adjusted by subtracting the mean of the 3 measurements taken at –1, –0.5, and 0 hours. Adjusted concentrations below 0 were set to 0. AUC_t_ was calculated using the linear trapezoidal method from time of administration until the last timepoint when the baseline-adjusted concentration was above 0. 

The parameters AUC_t_ and C_max_ for FSH were analyzed separately using a multiplicative analysis of variance model (ANOVA) (i.e., AUC_t_ and C_max_ were log-transformed before analysis) with sequence, subject within sequence, period, and treatment as factors. From this model, geometric mean AUC_t_ and C_max_ treatment ratios were estimated, and 90% confidence intervals for these were calculated. If the 90% confidence intervals for both parameters were within the bioequivalence limits of 0.8000 – 1.2500, it was concluded that the two formulations were bioequivalent. AUC_t_ and C_max_ for hCG were compared using the same method as for the primary endpoints. 

## Results 

### Subjects 

In total, 90 female subjects were screened, 76 women were randomized to a treatment sequence, and 56 women completed the trial for both treatment periods. Women who completed the trial had at screening a mean age of 29.0 years (range 20 – 40), a body weight of 64.2 kg (range 47 – 90), and a BMI of 22.8 kg/m^2^ (range 19 – 30). All women were White with the exception of 1 woman of Hispanic/Latino ethnicity. 

In total, 20 women were discontinued before starting treatment period 2; 3 women discontinued for personal reasons, 1 woman was discontinued on gynecologist recommendation, 1 woman due to use of prohibited medication, 2 women due to adverse events (polyfollicular ovaries and leukopenia, respectively), and 13 women were discontinued prior to treatment period 2 because of insufficient pituitary suppression on day –3 and/or day –1 prior to HP-hMG administration. These 13 women were sufficiently downregulated when starting the first treatment period, but their serum FSH levels increased and exceeded the protocol stipulated 5 IU/L limit by the start of the second treatment period. In contrast, their serum estradiol levels were well below the set criterion (≤ 50 pg/mL) and did not increase, indicating that the pituitary-gonadal axis of these women was profoundly suppressed. 

In treatment period 1, 39 women received HP-hMG liquid, and 37 women received HP-hMG powder, whereas in treatment period 2, 25 women received HP-hMG liquid, and 31 women received HP-hMG powder. 

### Pharmacokinetic evaluation of FSH 

**Figure 1.**
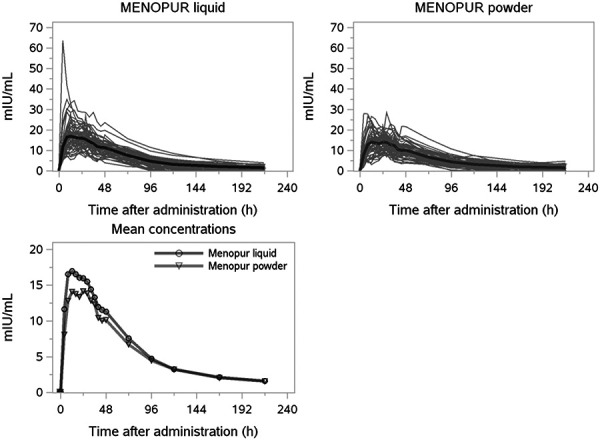
Individual and mean serum FSH concentrations.

**Figure 2.**
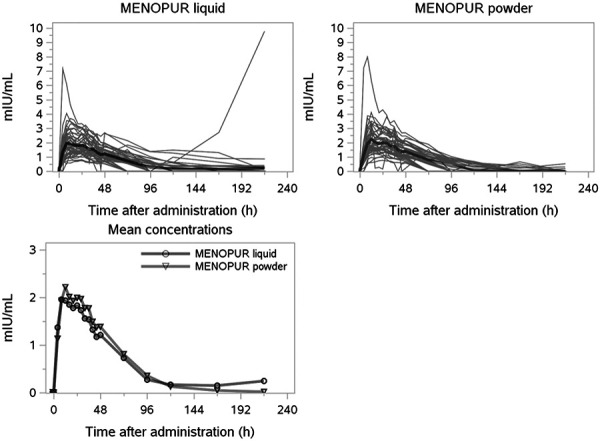
Individual and mean serum hCG concentrations.

Individual and mean serum FSH concentrations after administration of HP-hMG liquid and HP-hMG powder are presented in Figure 2. One woman included in the analysis had a relatively high peak serum FSH level (63.71 mIU/mL corrected for the endogenous baseline) at 4 hours after injection of HP-hMG liquid (Figure 1) and probably accidentally received at least part of the dose into a vein. In 1 additional woman, the peak serum FSH level occurred in the first post-dose sample at 4 hours after injection, in this case after administration of HP-hMG powder. The mean pre-dose serum FSH level was 3.36 mIU/mL, and values at the 3 pre-dose measurements at –1 hour, –0.5 hours, and just prior dosing were stable around this mean. 58 out of 336 pre-dose serum FSH measurements were below the lower limit of quantification (< 1.47 mIU/mL), as were 3 FSH measurements at 216 hours after dosing. 

Following a single subcutaneous dose of 450 IU HP-hMG, the geometric mean AUC_t_ of FSH was 1,296 h×mIU/mL (CV = 31%) for HP-hMG liquid and 1,143 h×mIU/mL (CV = 34%) for HP-hMG powder (Table 1). Mean peak serum FSH levels (C_max_) were 18.2 mIU/mL (CV = 35%) for HP-hMG pen and 15.6 mIU/mL (CV = 32%) for HP-hMG powder. The analysis of the primary endpoints AUC_t_ and C_max_ for FSH resulted in geometric mean ratios and 90% confidence intervals of HP-hMG liquid versus HP-hMG powder of 1.12 (1.0562 – 1.1889) for AUC_t_ and 1.17 (1.0946 – 1.2490) for C_max_ (Table 2). 

Other PK parameters for FSH (AUC_inf_, t_max_, T_1/2_, and CL/F) were comparable after administration of HP-hMG liquid and HP-hMG powder (Table 1). Maximal serum FSH concentrations were reached at 16 hours for HP-hMG liquid and 19 hours for HP-hMG powder, and terminal half-lives were 52 hours and 54 hours, respectively. AUC_t_ covered less than 80% of AUC_inf_ in 9 of 112 serum FSH PK profiles, with > 75% of AUC_inf_ covered in 5 of these 9 cases. 

### Pharmacokinetic evaluation of hCG 

245 of 300 pre-dose serum hCG concentrations were below the lower limit of quantification (< 0.500 mIU/mL). Individual and mean serum hCG concentrations after administration of 450 IU HP-hMG liquid and 450 IU HP-hMG powder are presented in Figure 2. The geometric mean AUC_t_ of hCG was 103 h×mIU/mL (CV = 71%) for liquid and 111 h×mIU/mL (CV = 58%) for powder, and the mean C_max_ was 2.04 mIU/mL (CV = 51%) and 2.16 mIU/mL (CV = 43%), respectively (Table 3). The mean AUC_inf_ of hCG was 82.3 h×mIU/mL (CV = 29%) for liquid and 84.2 h×mIU/mL (CV = 29%) for powder. The peak serum hCG level occurred in the first post-dose sample at 4 hours after injection in a single PK profile. AUC_t_ covered less than 80% of AUC_inf_ in 60 of 99 serum hCG PK profiles, with on average 73.9% of AUC_inf_ covered. In this study, AUC_inf_ of hCG was a less reliable measure of total exposure than AUC_t_. 

### Safety evaluation 

There were no serious adverse events reported, and no differences were noted in the incidence of adverse events between the two HP-hMG formulations (84.4% and 80.9%) or adverse drug reactions (48.4% and 51.5%). The most commonly reported adverse drug reactions (≥ 10% of women per treatment) were headache, injection-site reactions, and hot flush. Adverse events led to discontinuation of 2 women (2.9%) after administration of HP-hMG powder; 1 subject was diagnosed with multifollicular development, and 1 subject was diagnosed with leukopenia. No clinically significant changes in mean values of clinical chemistry and urinalysis variables, and no obvious treatment effects were observed from measurements of vital signs and ECG. All injection-site reactions were of mild intensity and were reported at a frequency of 20.3% and 64.7% after administration of HP-hMG liquid and HP-hMG powder, respectively. Among injection-site reactions, pain was reported at a frequency of 6.3% and 50.0% after administration of HP-hMG liquid and HP-hMG powder, respectively. 

## Discussion 

This trial demonstrated for the first time that HP-hMG (Menopur) in a liquid formulation administered with a pre-filled pen and 31G needle is bioequivalent to HP-hMG powder administered by conventional syringe. 

In the current trial, a relatively high dose of 450 IU HP-hMG was administered in accordance with the European guideline for bioequivalence studies [22]. In line with previous PK studies of menotropins in the dose range 225 – 445 IU [5], this bioequivalence trial showed a slow absorption from the injection site (t_max_ 19 hours) and a terminal half-life of ~ 2 days. Serum FSH levels were considerably higher in the current trial, also after adjusting for the higher menotropin dose. Such a difference may be related to different immunoassays and standards applied in these trials [23]. 

To prevent interference by endogenous FSH during the trial, female subjects were down-regulated with triptorelin, such that their endocrine status mimics those of IVF patients at the start of stimulation treated in a long GnRH agonist protocol. Administration of GnRH agonist as depot formulation is a long-established method to establish pituitary down-regulation of endogenous gonadotropins after a flare-up phase of 1 – 2 weeks [24]. GnRH agonists have successfully been used for this purpose in numerous PK studies [25, 26, 27, 28, 29]. Although it has been documented that GnRH agonist depot provides the most profound suppression of endogenous FSH, LH, and estradiol [30, 31], in the current trial, 13 women had to be discontinued from the trial due to insufficient FSH suppression prior to treatment period 2 following 3 triptorelin injections. Triptorelin depot is known to induce profound pituitary suppression leading to serum FSH levels of ~ 4 IU/L (range < 1 – 7 IU/L) following 14 days of treatment [30]. However, GnRH regulates the secretion of LH to a greater extent than that of FSH, which is known to be constitutively secreted [32]. Upon administration of triptorelin depot every 28 days in women with benign gynecologic disorders, Filicori et al. [31] report that triptorelin has at 14 days after the first administration a strong suppressive effect, followed by an increase in pre-dose FSH levels to mean values of 3.7 ± 0.4 IU/L on treatment day 196. The mean estradiol levels indicated profound pituitary suppression throughout the 6-month study period. In the current trial, the discontinuation of 13 women due to serum FSH levels > 5 IU/L at the start of treatment period 2 was not foreseen and is most probably related to a small increase of serum FSH levels over time, which may be related to the injections of HP-hMG being separated by 19 – 21 days in this study. 

In the current trial, hCG data from 6 women were excluded from the analysis as they had abnormally high serum hCG values that did not represent circulating hCG. Their hCG concentrations were unphysiologically high in all samples pre- and post-dose and across both treatment periods, with average levels of 15.1 mIU/mL (range 5.88 – 36.0 mIU/L). Therefore, the high signals are thought to be caused by interference in the hCG immunoassays, most likely by heterophile antibody interferences most frequently associated with the presence of human anti-mouse IgG antibodies (HAMA) in normal non-pregnant women and in men [33]. In the immunoassay used for hCG quantification, both capture and detection antibodies were mouse anti-hCG antibodies, which may have increased the risks for interference with HAMAs in the samples. The prevalence of HAMAs in normal subjects and specific investigations for samples with suspected heterophile antibody interference, such as HAMA, are described in the literature [34]. 

The development of an HP-hMG pre-filled multi-dose pen with characteristics comparable to other pens for treatment of diabetes or infertility is a logical step forward. Clinical practice has demonstrated that this type of device is more accurate, safer, and easier for patients, who are more compliant in administering the correct dose [20]. Accurate dose delivery is important during infertility therapy to prevent underdosing or overdosing. Errors may occur when reconstituting a lyophilized powder and administering with the conventional syringes, which affects the efficacy and safety of gonadotropin treatment. Apart from more accurate dose delivery, pen devices also have a finer and shorter needle than conventional needles applied with syringes, which improves the local tolerance [35]. 

In the current 2-way crossover trial, the overall adverse event profile was comparable between HP-hMG liquid and HP-hMG powder. Injection-site reactions were all mild and mostly reported as pain at the injection site probably because the total injection volume and needle of the pen were smaller than the injection volume and needle of the conventional syringe applied to inject the powder. 

## Conclusion 

In conclusion, this is the first report of a stable HP-hMG liquid formulation delivered to pituitary-suppressed women of reproductive age that is shown to be bioequivalent to HP-hMG powder and is well-tolerated. These findings support the future efficacy and safety of the HP-hMG pens in patients undergoing infertility treatment. 

## Ethnicity 

Due to the crossover study design, ethnicity is unlikely to affect the study conclusions. 

## Authors’ contributions 

DMJ, PL, AR, and BM designed the study; MK and RS conducted the study; BBR performed bioanalysis; DMJ and PL analyzed the data; DMJ and BM wrote the manuscript. 

## Funding 

This study was funded by Ferring Pharmaceuticals (Copenhagen, Denmark). 

## Conflict of interest 

DMJ, PL, AR, BBR, and BM are employees of Ferring Pharmaceuticals. 

**Table 1.** Pharmacokinetic parameters for FSH.

	MENOPUR powder (N = 56)	MENOPUR liquid (N = 56)
AUC_t_ (h×mIU/mL)		
Geometric mean (%CV)	1,142.75 (34.07)	1,296.06 (30.80)
Mean (SD)	1,201.90 (371.210)	1,352.78 (396.016)
min; max	485.00; 2316.51	547.50; 2802.91
AUC_inf_ (h×mIU/mL)		
Geometric mean (%CV)	1,300.57 (36.36)	1,433.98 (31.55)
Mean (SD)	1,375.45 (439.725)	1,499.75 (448.211)
min; max	495.34; 2578.51	630.67; 3,023.080
C_max_ (mIU/mL)		
Geometric mean (%CV)	15.55 (32.10)	18.19 (35.49)
Mean (SD)	16.30 (5.060)	19.40 (8.278)
min; max	7.83; 28.51	9.43; 63.71
CL/F (L/h)		
Geometric mean (%CV)	0.35 (36.36)	0.31 (31.55)
Mean (SD)	0.37 (0.154)	0.33 (0.110)
min; max	0.17; 0.91	0.15; 0.71
T_1/2_ (h)		
Geometric mean (%CV)	54.32 (43.99)	52.35 (30.63)
Mean (SD)	59.15 (26.381)	54.63 (15.939)
min; max	15.78; 183.76	26.29; 108.97
t_max_ (h)		
Geometric mean (%CV)	19.34 (50.71)	16.22 (56.76)
Mean (SD)	21.29 (8.254)	18.44 (9.047)
min; max	4.05; 36.02	4.03; 47.98

Based on baseline-adjusted serum concentrations.

**Table 2.** Analysis of AUC_t_ and C_max_ of FSH.

Endpoint	MENOPUR liquid geometric mean	MENOPUR powder geometric mean	Ratio	90% confidence interval
AUC_t_ (h×mIU/mL)	1,294.03	1,154.76	1.12	(1.0562; 1.1889)
C_max_ (mIU/mL)	18.29	15.65	1.17	(1.0946; 1.2490)

ANOVA on log-transformed endpoint with sequence, subject within sequence, period, and treatment as fixed factors.

**Table 3.** Pharmacokinetic parameters for hCG.

	MENOPUR powder	MENOPUR liquid
AUC_t_ (h×mIU/mL)	N = 50	N = 50
Geometric mean (%CV)	110.52 (58.35)	103.46 (70.88)
Mean (SD)	125.54 (59.437)	125.92 (85.695)
min; max	27.93; 262.92	24.48; 483.19
AUC_inf_ (h×mIU/mL)	N = 50	N = 49
Geometric mean (%CV)	150.42 (36.85)	142.64 (46.02)
Mean (SD)	159.70 (54.676)	157.01 (75.053)
min; max	52.74; 288.33	50.36; 473.57
C_max_ (mIU/mL)	N = 50	N = 50
Geometric mean (%CV)	2.16 (43.40)	2.04 (50.82)
Mean (SD)	2.36 (1.139)	2.32 (1.500)
min; max	0.77; 7.99	0.79; 9.78

Based on baseline-adjusted serum concentrations.
